# Adverse childhood experiences and sarcopenia: a prospective study embedded in the Canadian Longitudinal Study on Aging

**DOI:** 10.1093/ageing/afag050

**Published:** 2026-03-16

**Authors:** Menelaos M Dimitriadis, Kitty J Kokkeler, Emiel O Hoogendijk, Radboud M Marijnissen, Ivan Aprahamian, Hans W Jeuring, R C Oude Voshaar

**Affiliations:** University of Groningen, University Medical Center Groningen, Department of Psychiatry, Groningen, The Netherlands; Department of Old Age Psychiatry, Pro Persona Groep, Wolfheze, GE, The Netherlands; Amsterdam UMC Locatie VUmc—Department of Epidemiology & Data Science, Amsterdam Public Health Research Institute, P.O. Box 7057, Amsterdam 1081 HV, The Netherlands; University of Groningen, University Medical Center Groningen, Department of Psychiatry, Groningen, The Netherlands; Faculdade de Medicina de Jundiaí—Internal Medicine, Jundiaí, SP, Brazil; University of Groningen, University Medical Center Groningen, Department of Psychiatry, Groningen, The Netherlands; University of Groningen, University Medical Center Groningen, Department of Psychiatry, Groningen, The Netherlands

**Keywords:** sarcopenia, adverse childhood experiences, depression, aged, muscle mass, older people

## Abstract

**Background:**

Adverse childhood experiences (ACEs), known to increase lifelong health risks, have recently been linked to frailty. This study examined whether ACEs predict the onset and progression of sarcopenia, a core component of the frailty phenotype.

**Methods:**

We analysed 23 476 participants aged 45–85 years in the Canadian Longitudinal Study on Aging with 3-year follow-up (49.5% female; mean age 62.1 ± 9.9). Eight ACEs were assessed using validated retrospective instruments. Associations between ACE count and incident sarcopenia (revised European Working groups on Sarcopenia in Older People criteria) were evaluated using multivariable logistic regression, while changes in continuous sarcopenia components were assessed with lagged linear models adjusted for age, sex and ethnicity. Moderating effects of age, sex, ethnicity, depression (CESD ≥ 10) and socioeconomic position (education, income) were explored.

**Results:**

Overall, 12.7% of participants reported a high ACE burden (≥3). Among 21 910 participants without baseline sarcopenia, 614 (2.8%) developed sarcopenia. Depression, but not sociodemographic factors, moderated the ACE–sarcopenia association. ACE count predicted incident sarcopenia among depressed participants [OR (odds ratio) = 1.10 (95% (confidence interval) CI: 1.02–1.20), *P* = .016], but not among nondepressed participants [OR = 0.98 (95% CI: 0.93–1.03), *P* = .399]. Depression also moderated ACE-related declines in lean muscle mass and handgrip strength. Post hoc analyses showed that ACE-related worsening of chair rise performance and gait speed was mediated by depression.

**Conclusions:**

ACEs are associated with modest declines in muscle mass and function, with depression emerging as a key pathway linking early adversity to later-life sarcopenia. These findings highlight the need to integrate psychosocial history and mental health into risk stratification and preventive strategies for functional decline.

## Key Points

- Adverse childhood experiences (ACEs) are associated with incident sarcopenia in later life.
- Depression modifies the association between ACE and incident sarcopenia.
- Depression mediates the association between ACE and gait speed respective chair rise performance.
- Integration of psychosocial history and mental health is needed into risk stratification and prevention of functional decline.

## Introduction

Adverse childhood experiences (ACEs) are defined as traumatic experiences of people before the age of 18 that have lasting consequences for their somatic and mental health [1]. Overwhelming evidence shows that ACEs are among the strongest predictors of psychiatric disorders [2]. More recently, associations have been reported between ACE and several adverse physical health outcomes, like cardiovascular disease and invalidity [3, 4]. Hypothesised pathways include enduring effects of ACE on stress-related pathophysiological pathways (e.g. low-grade inflammation and hypercortisolism), on health-related behaviour (e.g. low physical activity), as well as the onset of mental disorders [2–4]. Moreover, these pathways are not mutually exclusive and may also contribute to the development of sarcopenia [5].

Sarcopenia refers to the combined loss of muscle strength and muscle mass and affects 10 to 27% of adults over 60 years of age [6, 7]. In 2016, sarcopenia received an ICD-10-CM code by the World Health Organization, acknowledging this condition as a disease state [8]. Sarcopenia is a multifactorial disease that increases the risk of falls, functional limitations, cardiovascular and respiratory conditions, cognitive impairment and prolonged hospitalisation, thereby substantially reducing health-related quality of life [8, 9]. Uncovering its determinants is therefore essential for guiding prevention and intervention strategies.

Recently, studies have identified ACE as an independent risk factor for physical frailty and its progression in later life [10–12]. Frailty reflects multisystem dysregulation and is typically operationalised through broad phenotypic or deficit-accumulation indices that capture overall physiological vulnerability [13]. Although frailty and sarcopenia share several biomarkers [14], sarcopenia represents only one, albeit important, pathway within the wider frailty spectrum [9, 15, 16]. Sarcopenia is a more specific construct, driven predominantly by neuromuscular degeneration, anabolic resistance and hormonal or inflammatory changes and is assessed using muscle-focused criteria. Correspondingly, sarcopenia is primarily targeted through resistance training and protein- or anabolic-based interventions, whereas frailty requires multicomponent strategies that address comorbidity, mobility, cognition and functional reserve [17]. Within the Canadian Longitudinal Study of Aging (CLSA), however, we did not find any association between ACE and baseline sarcopenia among circa 25 000 older adults [18]. Since cross-sectional studies may be biased by unknown selection processes (survival bias, memory bias, etc.), prospective studies are needed. So far, the longitudinal association between ACE and sarcopenia has only been examined once, among 6859 Chinese older adults [19]. In that study, a positive association between ACE and the onset of sarcopenia was observed, while social participation moderated this association [19].

In the current study, we further investigate factors that may modify the association between ACE and sarcopenia. This association may vary by age, sex and ethnicity, all of which are independently associated with ACE and sarcopenia [20–24]. Given the strong link between ACE and adult depression, and the well-established role of depression as a risk factor for sarcopenia through multiple biological and behavioural pathways [25, 26], depression is evaluated as a potential moderator and mediator. In addition, ACEs have been shown to adversely affect educational attainment and subsequent socioeconomic position (SEP), making SEP a further plausible pathway in this association [27]. Because ACEs are highly prevalent and unequally distributed, evaluating effect modification is essential from a public health perspective, as it may help to identify subgroups most vulnerable to the long-term physiological impact of early adversity and thus most likely to benefit from targeted prevention.

We hypothesise that individuals with ACE are more likely to develop sarcopenia in later life. Furthermore, we seek to examine whether the association between ACE and sarcopenia differs by age, sex, ethnicity, depression and socio-economic position.

## Method

### Sampling and design

**Figure 1:**
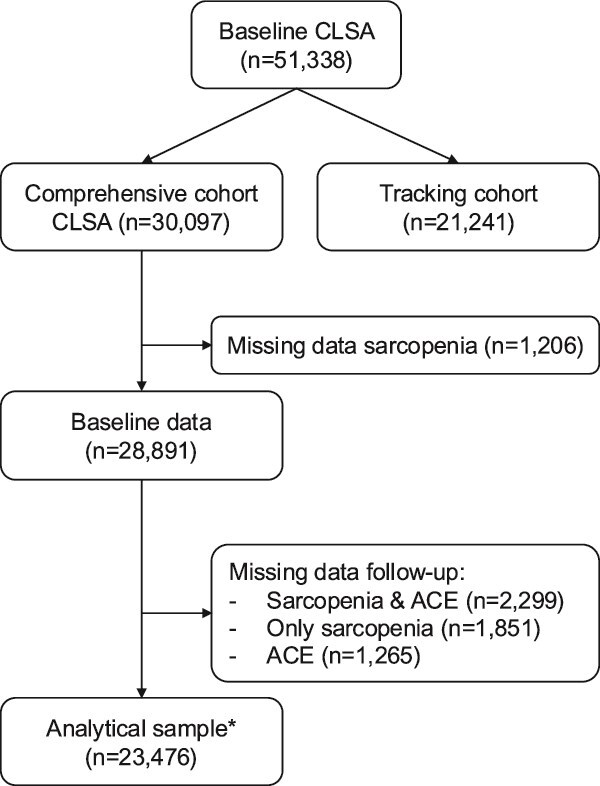
Flowchart of patient selection. Excluded participants were slightly older, more often female and non-White, had lower educational attainment and household income, smoked more, consumed less alcohol, were less physically active, reported higher depressive symptoms and more chronic diseases and showed a less favourable profile on sarcopenia-related measures (see Appendix 1, online).

This study was embedded in the CLSA. The CLSA recruited 51 338 people aged 45–85 years representative for Canada (CLSA cohort) [28, 29]. Of these, a randomly selected subsample of 30 097 living within 25–50 km of data collection sites formed the Comprehensive Cohort (in person assessment, including sarcopenia), whereas the remaining participants constituted the Tracking Cohort (telephone interviews and mailed questionnaires). For the present study, which used baseline (2010–15) and 3-year follow-up data (2014–18), we included all participants who completed the 3-year follow-up, as ACEs were assessed at that wave. Participants with missing data on sarcopenia at either baseline or follow-up were subsequently excluded, resulting in a final analytical sample of 23 476 (see Figure 1 and Appendix 1 for comparison of included and excluded participants).

### Outcome parameters (sarcopenia)

Sarcopenia (primary outcome) was defined according to the revised European Working Group on Sarcopenia in Older People (EWGSOP2) criteria [6]. Handgrip strength (kilograms), chair rise time (seconds) and appendicular lean muscle mass derived from dual X-ray absorptiometry (DXA) (kilograms, adjusted for height) were assessed at baseline and follow-up. The DXA machine was calibrated daily using a spine phantom, weekly using a whole-body step phantom, and yearly using a gold standard phantom [28]. Each component was assessed continuously and subsequently dichotomised using EWGSOP2 thresholds:

- Decreased muscle strength (yes/no): Participants were classified as having decreased strength if they scored below the sex-specific cut-off for either handgrip strength [maximum value from three attempts using a Wireless Grip Dynamometer (JTech Tracker Freedom)] or the chair rise test (time required to complete five rises) [28].
- Decreased muscle quantity (yes/no): Lean muscle mass was dichotomised using height-adjusted cut-off values from EWGSOP2 [28].

A binary sarcopenia variable was created: participants were classified as sarcopenic (yes = 1) if they showed both decreased muscle strength and decreased muscle mass; those meeting only one or neither criterion were classified as nonsarcopenic (no = 0).

We examined the individual components of the definition of sarcopenia, i.e. handgrip strength (kg), chair rise test (seconds) and lean muscle mass (kg), as secondary outcome parameters. We also included gait speed as a proxy for sarcopenia [30]. These variables were all collected as continuous variables at baseline and follow-up.

### Adverse childhood experiences

Each ACE item from the Childhood Experiences of Violence Questionnaire (CEVQ) and the Add Health Wave III instrument was first coded as a binary indicator (0 = no exposure, 1 = exposure), following established scoring rules [31]. Full item wording, coding rules and categorisation procedures are presented in Appendix 2.

Given the well-established dose–response relationship between the number of ACE score and adverse health outcomes, irrespective of the specific ACE types involved, and consistent with previous CLSA publications [31], we used the cumulative ACE count (range 0–8) as the primary exposure variable. In addition, we constructed an ordinal variable (0, 1, 2 or ≥ 3 ACEs) to facilitate comparisons between defined exposure categories, particularly between individuals with no ACE and those with high exposure (≥3).

### Covariates and effect modifiers

As potential confounders, we included age (years), sex (male/female) and Caucasian ethnicity (yes/no), which are all associated with either ACE or sarcopenia [22, 24, 32, 33].

Age, sex and ethnicity were also explored as potential effect-modifiers, in addition to depression and socio-economic position. Depression was measured at baseline and defined as a score of 10 or higher on the abbreviated version of the Centre of Epidemiologic Studies Depression (CESD) self-report questionnaire [34]. Socio-economic position was operationalised as educational attainment and equivalised household income. Educational attainment was categorised in four levels: lower (less than secondary school graduation), middle, bachelor level or master level and beyond. The equalised household income was calculated by taking the midpoint of each total household income category (defined as the total income of all household members, from all sources, before taxes and deductions, in the past 12 months, categories were (i) less than $20.000, (ii) $20.000 or more, but less than $50.000, (iii) $50.000 or more, but less than $100.000, (iv) $100.000 or more, but less than $150.000, (v) $150.000 or more), which was then divided by the square root of the total members of the household [35]. Finally, this result was categorised into tertiles representing low, medium and high-income groups.

### Statistical analyses

Multiple imputation was performed in SPSS (version 30, IBM Corp; Armonk, NY) using fully conditional specification to address missing data. Ordered categorical variables were recoded as continuous, and sparse categories were merged to ensure model stability. The imputation model included all variables used in the planned analyses as independent variables (predictors), supplemented with auxiliary variables associated with missingness (i.e. body mass index, alcohol use, physical activity and number of chronic diseases). Variables without missing values were included as complete predictors only (i.e. age, sex, ethnicity). A total of 20 datasets were generated, and estimates were pooled using Rubin’s rules.

Among participants not sarcopenic at baseline, we performed multivariable logistic regression analyses to examine the association between ACE count (independent variable) with the onset of sarcopenia at follow-up (dependent variable) adjusted for age, sex and ethnicity. Effect modification by age, sex, ethnicity, depression and socio-economic position was tested by their interaction terms with ACE count. Due to its categorical nature, educational attainment and equivalised household income were included as dummy variables. When significant, analyses were repeated in stratified groups. Sensitivity analyses were conducted by replacing ACE count by the predefined ordinal variable with participants with no ACE as reference [21].

Finally, lagged linear regression analyses were performed to investigate associations of ACE count with changes in the continuous components of sarcopenia (grip strength, gait speed, lean muscle mass, chair rise time). Since these analyses examined the progression of dimensional features over time, we did not exclude sarcopenic people at baseline. In each analysis, the follow-up value of the continuous sarcopenia component was the dependent variable and its baseline (i.e. lagged) value was included as an independent variable next to age, sex and ethnicity.

*P*-values <.05 were considered statistically significant.

## Results

**Figure 2:**
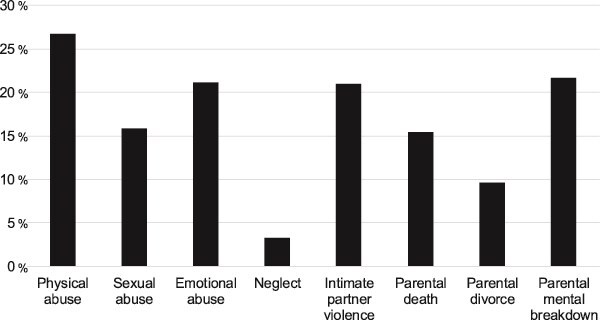
Proportion of the specific types of adverse childhood experiences at baseline (*n* = 23 476).

A total of 1566 out of the 23 476 participants (6.7%) fulfilled the criteria for sarcopenia at baseline. Table 1 depicts the characteristics of the whole study sample as well as the nonsarcopenic subgroup. Figure 2 presents the proportion of each subtype of ACE in the whole sample.

**Table 1:** Baseline characteristics of the whole study sample and nonsarcopenic sample at baseline

		Whole sample	Nonsarcopenic subsample
Characteristics:		(*n* = 23 476)	(*n* = 21 910)
Age (years)	mean (SD)	62.1 (9.9)	61.6 (9.7)
Female sex	*n* (%)	11 710 (49.9)	10 617 (48.5)
Caucasian ethnicity (yes)	*n* (%)	22 556 (96.1)	21 077 (96.2)
Level of highest education:			
Lower education	*n* (%)	4833 (20.6)	4438 (20.3)
Middle education	*n* (%)	6480 (27.6)	5957 (27.2)
Bachelor level	*n* (%)	6385 (27.2)	6021 (27.5)
Master level	*n* (%)	5788 (24.7)	5494 (25.1)
Income:			
Low	*n* (%)	7853 (33.5)	7046 (32.2)
Middle	*n* (%)	8299 (35.4)	7790 (35.6)
High	*n* (%)	7324 (31.2)	7074 (32.3)
Depression (CESD≥10)	*n* (%)	3273 (13.9)	2984 (13.6)
ACE count (0–8)	mean (SD)	1.4 (1.5)	1.4 (1.4)
Lean muscle mass (kg)	mean (SD)	21.4 (5.6)	21.8 (5.5)
Handgrip strength (kg)	mean (SD)	35.7 (11.8)	36.4 (11.6)
Gait speed (time in seconds)	mean (SD)	4.2 (0.9)	4.2 (0.9)
Chair rise test (time in seconds)	mean (SD)	13.2 (3.7)	12.9 (3.4)

### Primary outcome

A total of 614/21 910 (2.80%) nonsarcopenic participants developed sarcopenia at follow-up. Among nonsarcopenic participants (*n* = 21 910), ACE count was not associated with incident sarcopenia (Table 2). ACE count did not statistically significantly interact with age (*P* = .143), sex (*P* = .135), ethnicity (*P* = .359), educational attainment (middle, *P* = .252, bachelor, *P* = .683, master, *P* = .574) or equivalised household income (middle, *P* = .819, high, *P* = .930). However, the interaction between ACE count and current depression was statistically significant [B(SE) = 0.194 (0.067), *P* = .004]. Therefore, results were stratified by depression status.

**Table 2:** Association of adverse childhood experiences (ACE) with onset of sarcopenia at 3-year follow-up in the nonsarcopenic people at baseline applying logistic regression (*n* = 21 910)

				Stratified by depression					
	Whole sample^a^			No			Yes		
	OR	95% CI	*P*-value	OR	95% CI	*P*-value	OR	95% CI	*P*-value
Main model—ACE count (0–8):									
Age (years)	1.10	1.09–1.11	<.001	1.11	1.11–1.12	<.001	1.40	1.07–1.10	<.001
Female sex	1.60	1.36–1.89	<.001	1.80	1.58–2.06	<.001	1.40	1.04–1.88	.027
Non-Caucasian ethnicity	2.43	1.74–3.40	<.001	2.60	1.97–3.42	<.001	2.42	0.143–4.14	.001
ACE count (0–8)	1.03	0.97–1.09	.357	0.98	0.93–1.03	.399	1.10	1.02–1.20	.016
Sensitivity analyses^b^:									
No ACE (REF)	1.00			1.00			1.00		
One ACE	1.06	0.87–1.29	.571	1.03	0.89–1.20	.677	1.17	0.80–1.71	.416
Two ACEs	0.97	0.75–1.25	.784	1.00	0.82–1.22	.982	1.06	0.68–1.68	.788
Three or more ACEs	1.10	0.87–1.40	.418	0.93	0.76–1.14	.485	1.46	1.01–2.12	.045

Abbreviations: ACE, adverse childhood experiences; OR, odds ratio; CI, confidence interval.

^a^Adjusted for age, sex and ethnicity.

^b^Stratified models by depression.

A total of 524/18 926 (2.8%) nondepressed participants and 90/2984 (3.0%) depressed participants developed sarcopenia at 3-year follow-up. Table 2 shows that only among depressed participants, ACE count was associated with a greater incidence of sarcopenia at follow-up [odds ratio (OR) = 1.10 (95% confidence interval (CI): 1.02–1.20), *P* = .016)].

Sensitivity analyses using the ordinal classification of ACE (none, 1, 2 or ≥ 3 ACEs) showed that among depressed participants at baseline, those with ≥3 ACEs had a higher risk of incident sarcopenia than their counterparts with no ACE (OR = 1.46 (95% CI: 1.01–2.12), *P* = .045).

### Secondary outcome parameters

Regarding the secondary outcome parameters, we also found significant interactions between ACE count and depression status, but not with respect to age, sex, ethnicity, educational attainment or equivalised household income. ACE count significantly interacted with depression when predicting lean muscle mass at follow-up (*P* = .032), and grip strength (*P* = .012), but not when predicting changes in gait speed (*P* = .733) or chair rise test (*P* = .613).

As shown in Table 3, ACE count was borderline significantly associated with lower muscle mass at follow-up [B(SE) = −0.028 (0.015), *P* = .062] and significantly with a lower grip strength [B(SE) = −0.108 (0.046), *P* = .019] in the depressed subsample, but not among nondepressed participants. Moreover, in the entire sample, ACE was significantly associated with a longer time to perform the walking test (lower gait speed) (B(SE) = 0.003 (0.001), *P* < .001) and to perform the chair rise test (B(SE) = 0.004 (0.001), *P* < .001). The sensitivity analyses on the ordinal classification of ACE are presented in Supplementary Table 3 (see Appendix 3).

**Table 3:** Association of ACE with proxies for sarcopenia at 3-year follow-up (adjusted for their baseline values, age, sex and ethnicity)

	Lean muscle mass (kg)		Grip strength (kg)	
	B (SE)	*P*-value	B (SE)	*P*-value
Stratified analyses (by depression status)				
Nondepressed subgroup				
ACE count (0–8)	0.008 (0.007)	.218	0.019 (0.023)	.838
Depressed subgroup				
ACE count (0–8)	−0.028 (0.015)	.062	−0.108 (0.046)	.019
	Gait speed (4 m walking time)(s)		Chair rise test (time) (s)	
	B (SE)	*P*-value	B (SE)	*P*-value
Whole sample				
ACE count (0–8)	0.003 (0.001)	<.001	0.004 (0.001)	<.001

Abbreviations: ACE, adverse childhood experiences; SE, standard error; *n*, number of participants; 4 m, four metres; s, seconds.

### Post hoc analyses

Given the absence of a main effect of ACE count on incident sarcopenia, decline in lean muscle mass, and decline handgrip strength, but its presence among participants with depression (i.e. a moderation effect), we conducted post hoc analyses to examine whether the main effect of ACE count on gait speed and chair rise test in the total sample was mediated by baseline depressive symptom severity. Using the PROCESS macro (version 5) in SPSS, the indirect effect of ACE count through depression was significant for both outcomes [for gait speed: 0.0016 (95% CI: 0.0013–0.0019); for chair rise test: 0.0014 (95% CI: 0.0011–0.0018)]. The direct effect of ACE count was no longer significant for gait speed [0.0009 (95% CI: −0.0005–0.0024)], but remained significant for the chair rise test [0.0024 (95% CI: 0.0004–0.0043)]. Further details are provided in Appendix 4 (Supplementary Tables 4 and 5).

## Discussion

### Main findings

While separate studies have shown that ACE is associated with the onset of late life depression [36, 37], and late life depression with the onset of sarcopenia [26], this study shows that the association between ACE and onset and progression of sarcopenia in middle aged and older adults is conditional upon depression. ACE count increased the risk of incident sarcopenia and accelerated decline in muscle mass, grip strength, gait speed and chair-rise ability among individuals with depression, whereas associations were weaker or absent in their nondepressed counterparts. This suggests that early-life adversity may influence later-life physical functioning partly through its impact on mental health.

### Interpretation

Our study replicated known determinants of sarcopenia, including higher age, female sex, non-Caucasian ethnicity, lower educational attainment and lower income [38]. However, these associations do not necessarily imply that childhood adversity exerts differential effects across these subgroups. Biological embedding of adversity likely unfolds earlier in adulthood through educational, occupational, behavioural and physiological pathways, resulting in relatively uniform effects by mid-to-late life. Differential susceptibility to ACE may therefore be less detectable at older ages. Nonetheless, selective survival and cohort attrition may have reduced variability in older age groups, leading to conservative interaction estimates.

Depression, in contrast, may represent a more proximal indicator of vulnerability to the physiological consequences of ACE, such as inflammation and HPA-axis dysregulation [5, 39], which could explain why it emerged as a significant moderator whereas demographic factors did not. In this context, depression appears an important pathway linking childhood trauma to impairments in overall physical functioning, including mobility, balance and lower-extremity performance. Rather than pointing to isolated muscle or joint problems, the findings indicate a broader reduction in functional capacity that affects the ability to move efficiently and perform basic mobility tasks.

The finding that depression moderated ACE effects on muscle mass and strength but mediated performance-based decline may suggest differential pathways for these components of sarcopenia. Performance measures such as gait speed and chair rise rely more on lower-extremity function, balance and coordination, whereas muscle mass and grip strength reflect neuromuscular and anabolic capacity [40]. These distinctions may explain why depression influences ACE-related decline in performance differently than decline in muscle mass and strength.

Our findings extend the first and only prospective study on ACE and sarcopenia conducted in middle-aged and older Chinese people [19], showing that the association between ACE and incident sarcopenia generalises to Western populations. This is important as Asian populations generally have lower height-adjusted lean muscle mass compared to Caucasian populations [41] which could potentially impact the development and progression of sarcopenia. Therefore, assessment of sarcopenia in this previous study was based on the Asian Working Group for Sarcopenia 2019 algorithms. While both studies included people aged 45+ years and older, the prevalence of sarcopenia was still nearly twice as high among Chinese people (14.6%) compared to our Canadian population (6.7%). This difference in prevalence probably reflects ethnic differences. Nonetheless, we cannot rule out systematic bias, as our study used DXA scans, the gold standard for assessing appendicular lean muscle mass, whereas the Chinese study relied on estimates based on anthropometrics measurements [19].

Of interest was the moderation by social participation in the Chinese study and by depression in our sample. Depression and lack of social participation are consistently associated [42]. Therefore, the effect of ACE on somatic health might not be as prominent as thought [1, 11], while the robust detrimental impact of ACE on mental health [4, 43] may be a prominent pathway to sarcopenia.

### Methodological considerations

The most significant strength of this study is the longitudinal design in a large sample representative of middle-aged and older Canadian people. This allowed us to investigate our research questions with a higher degree of precision, resulting in more robust and reliable findings with a greater generalisability and external validity.

ACE research shows substantial heterogeneity in how adversity is defined and measured. Studies vary in the number and types of ACE included, how items are grouped (e.g. parental vs. personal adversity; abuse vs. household dysfunction), and whether ACEs are analysed cumulatively, categorically or by subtype. A limitation of this study is the retrospective assessment of ACE, which may introduce recall bias. Although prior work indicates that such bias is acceptable [19, 21, 44] and that ACEs remain robust predictors of adult health, differences in measurement still require that ACE–health associations be interpreted in the context of the specific assessment used [45]. Secondly, a follow-up period of 3 years might be too short to definitively assess the longitudinal effect of ACE on sarcopenia development as its incidence is relatively low and generally rises with age [46]. Thirdly, selection bias is inherent to large epidemiological studies. While the CLSA is generalisable to the Canadian population on many important variables, the Comprehensive Cohort, in particular, is more educated, has a higher income, is less often non-White ethnicity and rated their general health as very good [29]. Furthermore, the profile of study dropouts suggests selective dropouts among socio-economically disadvantaged groups. Since socio-economic position did not moderate or mediate outcome, selection bias might have been modest. Finally, covariates were selected parsimoniously to avoid overadjustment, as potential confounders such as lifestyle factors and somatic disease burden can also be conceptualised as mediators on the pathway from ACE to depression, from ACE to sarcopenia and from depression to sarcopenia. Future studies should examine these mediating processes across multiple waves, considering the bidirectional relationship with depression as a moderating factor.

## Conclusion

This study underscores the importance of incorporating psychosocial history into healthy-ageing strategies. Although follow-up was limited to 3 years, even modest effects may accumulate substantially over longer periods. Among older adults with depression, each additional ACE increased the odds of developing sarcopenia by 10%, translating into a 46% higher risk for those with three or more ACEs. Given the high prevalence and treatability of depression, these findings identify a feasible clinical and public health intervention point. Routine assessment of psychosocial history, including childhood adversity, screening for early functional decline, and the provision of targeted exercise, nutrition and mental health interventions may help prevent or delay sarcopenia in this vulnerable subgroup.

## Supplementary Material

aa-25-2492-File004_afag050

## Data Availability

Data are available from the CLSA (www.clsa-elcv.ca) for researchers who meet the criteria for access to de-identified CLSA data.
