# Supplementary material for: Adverse childhood experiences and sarcopenia: a prospective study embedded in the Canadian Longitudinal Study on Aging
Source: Age Ageing. 2026 Mar 16;55(3):afag050. doi: 10.1093/ageing/afag050 (PMC13016992; doi:10.1093/ageing/afag050)
Supplement: aa-25-2492-File004_afag050 [file aa-25-2492-file004_afag050.pdf]

Content list Supplementary Data:

Appendix 1 - Comparison of included and excluded CLSA participants

Appendix 2 - Measurement of ACE

Appendix 3 - Secondary outcome measures (extensive table)

Appendix 4 - Post-hoc mediation analyses

Appendix 1 – Comparison of included and excluded CLSA participants

Supplementary Table 1

Comparison of in- and excluded participants of the comprehensive CLSA cohort

**Characteristics**		**Included (23,476)**	**Excluded (6,621)**	**Statistics**
• Age (years)	mean (SD)	62.1 (9.9)	65.9 (10.9)	t=26.5, df=30,095, p<.001
• Female sex	n (%)	11,710 (49.9)	3,610 (54.5)	Chi^2=44.5, df=2, p<.001
• Caucasian ethnicity	n (%)	22,556 (96.1)	6,215 (93.9)	Chi^2=60.1, df=2, p<.001
• Leven of education				
○ Low	n (%)	4,094 (20.2)	1,475 (28.0)	Chi^2=187.5, df=3, p<.001
○ Medium	n (%)	5,037 (24.8)	1,374 (26,1)	
○ Bachelor	n (%)	5,818 (28.7)	1,258 (23.9)	
○ Master	n (%)	5,343 (26.3)	1,153 (21.9)	
• Equivalized household income				
○ Low	n (%)	7,420 (33.5)	2,918 (48.6)	Chi^2=509.1, df=2, p<.001
○ Medium	n (%)	7,861 (35.5)	1,878 (31.3)	
○ High	n (%)	6,866 (31.0)	1,213 (20.2)	
• Body mass index (kg/m2)	mean (SD)	28.0 (5.3)	28.2 (5.9)	t=1.7, df=29,951, p=.092
• Smoking				
○ Never	n (%)	11,404 (48.6)	2,832 (42.8)	Chi^2=137.4, df=2, p<.001
○ Ever	n (%)	10,171 (43.4)	2,971 (44.9)	
○ Current	n (%)	1,900 (8.1)	809 (12.2)	
• Alcohol use				
○ Less than monthly	n (%)	5,060 (22.0)	2,072 (32.4)	Chi^2=322.4, df=3, p<.001
○ 1 – 4 times a month	n (%)	6,529 (28.4)	1,737 (27.2)	
○ 2 – 3 times a week	n (%)	5,075 (22.1)	1,059 (16.6)	
○ ≥4 times a week	n (%)	6,313 (27.5)	1,526 (23.9)	
• Physical activity (PASE)	mean (SD)	380 (97)	292 (153)	t=-56.7, df=30,095, p<.001
• Depressive symptoms (CESD)	mean (SD)	5.0 (4.5)	6.3 (5.1)	t=20.6, df=29,934, p<.001
• Chronic disease (number)	mean (SD)	2.1 (1.8)	2.7 (2.2)	t=23.2, df=27,787, p<.001
• Muscle mass (kg)	mean (SD)	7.4 (1.1)	7.1 (1.4)	t=-15.2, df=28,780, p<.001
• Walking time 4m (s)	mean (SD)	4.1 (2.7)	4.5 (3.6)	t=10.1, df=29,719, p<.001
• Chair rise test (s)	mean (SD)	13.1 (4.4)	14.0 (5.7)	t=12.1, df=28,772, p<.001
• Grip strength (kg)	mean (SD)	35.9 (11.8)	32.2 (11.7)	t=-20.0, df=27,796, p<.001

Appendix 2 - Measurement of ACE

Adverse childhood experiences were assessed with the short form of the Childhood Experiences of Violence Questionnaire (CEVQ) (Walsch et al, 2008; Tanaka et al, 2012) and the National Longitudinal Study of Adolescent to Adult Health Wave III questionnaire (Harris & Udry, 2014). This resulted in a total of 14 items (see below) that all refer to exposure before the age of 16 years. Items relating to physical abuse, sexual abuse, emotional abuse, neglect, and intimate partner violence were assessed on an ordinal scale (never, 1–2 times, 3–5 times, 6–10 times or more than 10 times) and subsequently dichotomized as presence or absence of exposure based on the CEVQ instructions (Tanaka et al, 2012):

- Physical abuse was present if the participant reported being slapped on the face, head or ears, or hit or spanked with something hard 3 or more times; being pushed, grabbed or shoved, or having something thrown to hurt 3 or more times; or being kicked, bit or punched, or choked, burned or physically attacked in some other way 1 or more times.
- Sexual abuse was present if the participant reported being threatened, touched or forced into unwanted sexual activity 1 or more times.
- Emotional abuse was present if the participant reported parents or guardians swearing, saying hurtful or insulting things that made the participant feel unloved or unwanted 3 or more times.
- Childhood exposure to intimate partner violence was present if the participant reported seeing or hearing parents or guardians say hurtful things to each other 6 or more times, or seeing or hearing parents or guardians hit each other 3 or more times.
- Participants were classified as being neglected if they reported their parents or guardians not having taken care of their basic needs.

The three other ACEs, i.e. parental divorce or separation, parental death, or living with a family member with mental health problems, were assessed dichotomously.

As stated in Joshi et al (2021): "*Test reliability of the CEVQ has not been assessed in adults between the ages of 45 and 85 years. The 2-week test–retest reliability of the CEVQ short form (CEVQ-SF) in measuring physical and sexual abuse among youth were $\kappa = 0.61$ and $\kappa = 0.91$, respectively. Kappa values for other forms of abuse and household adversity ranged between 0.66 and 0.86 (Dube et al, 2004). The criterion validity of the CEVQ-SF in comparison to the Childhood Trauma Questionnaire was satisfactory (Tanaka et al, 2012). Construct validity was shown by observing a higher odds of clinical traumatic symptoms among physically and sexually abused individuals compared with either type alone (Tanaka et al, 2015).*

Given the well-established dose-response relationship between the number of ACEs score and adverse health outcomes, irrespective of the specific ACE types involved (Merrick et al, 2017; Voellmin et al, 2015; Gilbert et al, 2010), and consistent with previous CLSA publications (Joshi et al, CMAJ Open 2021), we used the cumulative ACE count (range 0 – 8) as the primary exposure variable. In addition, we constructed an ordinal variable (0, 1, 2, or ≥3 ACEs), allowing category-specific comparisons particularly between individuals with no ACEs and those with a high (≥3) exposure."

Box 1- Items adapted from the Childhood Experiences of Violence Questionnaire (CEVQ)

1. Before age 16, how many times did any one of your parents, stepparents or guardians swear at you, or say hurtful, insulting things that made you feel like you were not wanted or loved?
2. Before age 16, how many times did you see or hear any one of your parents, stepparents or guardians hit each other or another adult in your home? By adult, I mean anyone 18 years and over.
3. Before age 16, how many times did a parent or caregiver spank you with their hand on your bottom (bum), or slap you on your hand?
4. Before age 16, how many times did an adult slap you on the face, head or ears or hit or spank you with something hard to hurt you?
5. Before age 16, how many times did an adult push, grab, shove or throw something at you to hurt you?
6. Before age 16, how many times did an adult kick, bite, punch, choke, burn you, or physically attack you in some way?
7. Before age 16, how many times did your parents, stepparents or guardians not take care of your basic needs, such as keeping you clean or providing food or clothing?
8. Before age 16, how many times did an adult force you or attempt to force you into any unwanted sexual activity, by threatening you, holding you down or hurting you in some way?
9. Before age 16, how many times did an adult touch you against your will in any sexual way? By this, I mean anything from unwanted touching or grabbing, to kissing or fondling.
10. Before age 16, did you ever see or talk to the police or anyone from child protective services about any of the things you mentioned?
11. Before age 16, how many times did you see or hear any one of your parents, stepparents or guardians say hurtful or mean things to each other or to another adult in your home?
12. Did you ever experience the death or serious illness of a parent or a primary caretaker?
13. Did you experience the divorce or separation of your parents?
14. Did anyone in your family ever suffer from mental or psychiatric illness or have a "breakdown"?

Supplementary Table 2

Proportion of adverse childhood experiences, stratified by sarcopenia (yes/no) and stratified by depression status at baseline (CESD≥10, yes/no)

	**Total sample**	**Sarcopenia**		**Depression (CESD≥10)**	
**ACEs, Index, n(%)**		No	Yes	No	Yes
0	8802 (37.5)	8161 (37.2)	641 (40.9)	7905 (39.2)	866 (26.5)
1	6483 (27.6)	6008 (27.4)	475 (30.3)	5644 (28.0)	823 (25.2)
2	3570 (15.2)	3368 (15.4)	202 (12.9)	3033 (15.1)	530 (16.2)
3	2165 (9.2)	2054 (9.4)	111 (7.1)	1727 (8.6)	436 (13.4)
4	1314 (5.6)	1240 (5.7)	74 (4.7)	1010 (5.0)	300 (9.2)
5	708 (3.0)	667 (3.0)	41 (2.6)	519 (2.6)	187 (5.7)
6	308 (1.3)	292 (1.3)	16 (1.0)	222 (1.1)	86 (2.6)
7	105 (0.4)	99 (0.5)	6 (0.4)	74 (0.4)	30 (0.9)
8	21 (0.1)	21 (0.1)	0	15 (0.1)	5 (0.2)

References

- Walsh CA, MacMillan HL, Trocmé N, et al. Measurement of victimization in adolescence: development and validation of the Childhood Experiences of Violence questionnaire. Child Abuse Negl 2008;32:1037-57.
- Tanaka M, Wekerle C, Leung E, et al. Preliminary evaluation of the Childhood Experiences of Violence Questionnaire short form. J Interpers Violence 2012;27:396-407.
- Harris KM, Udry JR. National Longitudinal Study of Adolescent to Adult Health 1994-2008. Chapel Hill (NC): Carolina Population Center, University of North Carolina-Chapel Hill, Inter-university Consortium for Political and Social Research; 2014.
- Dube SR, Williamson DF, Thompson T, et al. Assessing the reliability of retrospective reports of adverse childhood experiences among adult HMO members attending a primary care clinic. Child Abuse Negl 2004;28:729-37.
- Merrick MT, Ports KA, Ford DC, et al. Unpacking the impact of adverse childhood experiences on adult mental health. Child Abuse Negl 2017;69:10-9.
- Voellmin A, Winzeler K, Hug E, et al. Blunted endocrine and cardiovascular reactivity in young healthy women reporting a history of childhood adversity. Psychoneuroendocrinology 2015;51:58-67.
- Gilbert LK, Breiding MJ, Merrick MT, et al. Childhood adversity and adult chronic disease: an update from ten states and the District of Columbia, 2010. Am J Prev Med 2015;48:345-9.

Appendix 3 – Secondary outcome measures (extensive table)

Supplementary Table 3

Association of ACE with proxies for sarcopenia at three-year follow-up (adjusted for their baseline values, age, sex, and ethnicity)

	**Whole sample (n=23,476)**		**Non-depressed subgroup (n=20,203)**		**Depressed subgroup (n=3,273)**	
	B (SE)	p-value	B (SE)	p-value	B (SE)	p-value
**Lean muscle mass**						
- ACE count (0-8)	0.002 (0.006)	.362	0.008 (0.007)	.218	-0.028 (0.015)	.062
*Sensitivity analysis**:*						
- One ACE	0.010 (0.023)	.427	0.013 (0.029)	.594	-0.018 (0.071)	.795
- Two ACEs	0.004 (0.027)	.147	-0.004 (0.029)	.878	0.037 (0.081)	.649
- Three or more ACEs	0.010 (0.025)	.416	0.024 (0.028)	.872	-0.066 (0.069)	.334
**Handgrip strength (kg)**						
- ACE count (0-8)	-0.018 (0.021)	.373	0.019 (0.023)	.838	-0.108 (0.046)	.019
*Sensitivity analysis**:*						
- One ACE	0.066 (0.076)	.385	0.049 (0.081)	.541	0.266 (0.219)	.226
- Two ACEs	0.094 (0.091)	.304	0.170 (0.098)	.083	-0.222 (0.249)	.373
- Three or more ACEs	-0.016 (0.086)	.852	0.104 (0.096)	.279	-0.229 (0.209)	.275
**Gait speed (time)**						
- ACE count (0-8)	0.003 (0.001)	<.001	0.002 (0.001)	.018	0.001 (0.002)	.484
*Sensitivity analysis**:*						
- One ACE	0.000 (0.003)	.889	0.001 (0.003)	.703	-0.18 (0.009)	.042
- Two ACEs	0.002 (0.003)	.554	0.000 (0.003)	.910	0.004 (0.010)	.679
- Three or more ACEs	0.011 (0.003)	<.001	0.008 (0.003)	.012	0.001 (0.008)	.893
**Chair rise test (time)**						
- ACE count (0-8)	0.004 (0.001)	<.001	0.003 (0.001)	.003	0.004 (0.002)	.117
*Sensitivity analysis**:*						
- One ACE	-0.003 (0.004)	.462	0.000 (0.004)	.909	-0.027 (0.011)	.016
- Two ACEs	0.014 (0.004)	.001	0.013 (0.005)	.006	0.006 (0.013)	.638
- Three or more ACEs	0.013 (0.004)	.001	0.009 (0.004)	.033	0.001 (0.011)	.951

* Adjusted for age, sex, and ethnicity
** Reference is “no ACE”

Appendix 4 - Post-hoc mediation analyses

The post-hoc mediation analyses were conducted using the PROCESS macro for SPSS version 5 (see https://afhayes.com/introduction-to-mediation-moderation-and-conditional-process-analysis.html) adjusted for age, sex, and ethnicity. Depression was included as a continuous measure (CES-D sum score). We applied bootstrapping (5000 samples) in the non-imputed analytical sample.

Supplementary Table 4

Direct and indirect effect through depression of ACE count on gait speed and chair rise test*

	Decline in gait speed**		Decline in Chair rise***	
**Effect of ACE count**	B (SE)	[95% C.I.]	B (SE)	[95% C.I.]
• Direct effect	.0009 (.0007)	[-.0005, .0024]	.0024 (.0010)	[.0004, .0043]
• Indirect effect through depression	.0016 (.0002)	[.0013, .0019]	.0014 (.0002)	[.0011, .0018]

* Models adjusted for age, sex, and ethnicity.

To provide additional information on the independent effect of depression, as well as socio-economic position (educational attainment and equivalised household income), we also present the lagged regression analyses adjusted for these variables in the imputed dataset (see supplementary table 5).

Supplementary Table 5

Independent effect of depression (10-item CESD sum score), educational attainment (lower = reference), or equivalised household income (lower = reference) on change in gait speed or chair rise including the unique contribution of ACE count with and without these covariates.

	**Gait speed** (time needed in seconds)				**Chair rise test** (time needed in seconds)			
	**B (SE)**	**t**	**p**	**ΔR^2**	**B (SE)**	**t**	**p**	**ΔR^2**
No potential mediator included:								
- ACE count (0 - 8)	0.003 (0.001)	3.6	<.001	.019	0.004 (0.001)	4.5	<.001	.025
Depression included:								
- CESD sum score	0.003 (0.000)	12.4	<.001	.065	0.003 (0.000)	10.3	<.001	.054
- ACE count (0 - 8)	0.001 (0.001)	1.4	.158	.007	0.003 (0.001)	2.7	.007	.015
Education included:								
- Middle	-0.010 (0.004)	-2.8	.006	-.016	-0.007 (0.004)	-1.6	.111	-.009
- Bachelor	-0.019 (0.003)	-5.7	<.001	-.032	-0.021 (0.004)	-4.7	<.001	-.027
- Master	-0.032 (0.003)	-9.4	<.001	-.052	-0.027 (0.004)	-6.1	<.001	-.034
- ACE count (0 - 8)	0.002 (0.001)	3.0	.002	.016	0.004 (0.001)	4.0	<.001	.022
Income included:								
- Middle	-0.022 (0.003)	-8.3	<.001	-.045	-0.019 (0.004)	-5.1	<.001	-.029
- Higher	-0.024 (0.003)	-8.4	<.001	-.046	-0.030 ()0.004	-8.0	<.001	-.045
- ACE count (0 - 8)	0.002 (0.001)	.20	.004	.015	0.004 (0.001)	3.9	<.001	.022

* Adjusted for age, sex, and ethnicity

Abbreviations: ACE, adverse childhood experiences; CESD, Center for Epidemiological Scale for Depression; SE, standard error; p, p-value; ΔR^2, unique variance by that variable.
